# Are Neurotrophin Genes Involved in the Pathophysiology of Gambling Disorder?

**DOI:** 10.1192/j.eurpsy.2022.621

**Published:** 2022-09-01

**Authors:** I. Baenas, N. Solé-Morata, M. Etxandi, R. Granero, M. Gené, C. Barrot, P. Gorwood, N. Ramoz, F. Fernandez-Aranda, S. Jimenez-Murcia

**Affiliations:** 1 Hospital Universitario de Bellvitge, Psiquiatría, L’Hospitalet de Llobregat, Spain; 2 Instituto de Salud Carlos III, Ciber Fisiopatología De La Obesidad Y Nutrición, Madrid, Spain; 3 Autonomous University of Barcelona, Department Of Psychobiology And Methodology, Bellaterra, Spain; 4 Laboratorio de Genética, Unidad de Medicina Legal y Toxicología, Facultad de Medicina, Universidad de Barcelona, Ciencias Clínicas, Barcelona, Spain; 5 Unisersité Paris Descartes, Cmme, Hopital Sainte-anne Ghu Paris Psychiatrie Et Neurosciences, Paris, France; 6 School of Medicine and Health Sciences, University of Barcelona, Department Of Clinical Sciences, Barcelona -Campus Bellvitge, Spain; 7 University Hospital of Bellvitge,-IDIBELL, Department Of Psychiatry, Hospitalet de Llobregat, Spain; 8 Instituto de Investigación Biomédica de Bellvitge (IDIBELL), Grupo Psiquiatría Y Salud Mental-programa Neurociencias, Hospitalet de Llobregat, Spain

**Keywords:** Neurotrophin genes, SNPs, Haplotypes, Gambling Disorder

## Abstract

**Introduction:**

Gambling Disorder (GD) is considered a multifactorial behavioral addictive disorder, leading to severe psychological, social and economic consequences. Previous studies have investigated genetic mechanisms underlying GD. Growing literature showed a possible link between addiction-related disorders and neurotrophic factors (NTF), involved in synaptic plasticity and neuronal survival. Thus, the study of NTF genes emerged as promising targets in the field of GD.

**Objectives:**

To evaluate genetic implications of the NTF family in the pathophysiology GD. We hypothesized the involvement of some NTF genes polymorphisms in the onset and progression of GD as potential biological risk factors.

**Methods:**

The sample was composed by 166 individuals with GD and 191 healthy controls. 36 Single nucleotide polymorphisms (SNPs) from NTF (NGF, NGFR, NTRK1, BDNF, NTRK2, NTF3, NTRK3, NTF4, CNTF and CNTFR) were selected and genotyped. Linkage disequilibrium and haplotype constructions were assessed, related to the presence of GD. Moreover, regulatory elements overlapping the identified SNPs variants associated with GD was also analyzed.

**Results:**

6 SNPs were potentially associated to GD after the comparisons of allele frequencies between groups. Single and multiple-marker analyses showed a strong association between both NTF3 and NTRK2 genes, and GD.

**Conclusions:**

This study suggests the implication of NTF genes in the development of GD, being the altered cross-regulation of some NTF factors signalling pathways, a potential biological vulnerability factor in GD. Fundings and Acknowledgements: Ministerio de Ciencia, Innovación y Universidades (RTI2018-101837-B-100) Delegación del Gobierno para el Plan Nacional sobre Drogas (2017I067, 2019I47), Instituto Salud Carlos III (ISCIII) (PI17/01167, PI20/00132) and CIBERObn, an initiative of ISCIII.

**Disclosure:**

No significant relationships.

